# Intelligent prosthetic hips and knees: from actuation to perception and control

**DOI:** 10.3389/fnins.2025.1690921

**Published:** 2026-01-12

**Authors:** Xiaoming Wang, Yuanhua Li, Hongliu Yu

**Affiliations:** 1 Institute of Rehabilitation Engineering and Technology, University of Shanghai for Science and Technology, Shanghai, China; 2 Shanghai Engineering Research Center of Assistive Devices Institute of Rehabilitation Engineering and Technology, Shanghai, China; 3 Key Laboratory of Neural-functional Information and Rehabilitation Engineering of the Ministry of Civil Affairs, Shanghai, China

**Keywords:** actuation mechanism, motion intention recognition, prosthesis control, prosthetic hip, prosthetic knee

## Abstract

Intelligent prosthetic hips and knees represent a critical advancement in restoring natural gait and mobility for lower-limb amputees, particularly those with high-level amputations such as hip disarticulation. This systematic review examines recent progress in three fundamental aspects of intelligent prosthetic technology: actuation, perception, and control. In terms of actuation, the review highlights the limitations of passive and active prostheses and discusses emerging hybrid active-passive mechanisms that aim to replicate the natural, biarticular muscle-driven energy transfer in human gait. The perception section addresses current methodologies for recognizing human motion intentions through mechanical, bioelectric, biomechanical, and external environmental signals, underscoring the challenges of stability, latency, and interference inherent in existing approaches. Regarding control strategies, the paper categorizes intelligent control into torque compensation, motion following, and direct intention control, outlining the strengths and limitations of each method. The review identifies critical technological bottlenecks, including signal interference, limited adaptability to dynamic environments, and the absence of effective real-time intention recognition methods. The paper concludes by suggesting future directions in the development of hybrid actuation and advanced perception-control integration, essential for improving the usability and efficacy of intelligent prosthetic hips and knees, ultimately enhancing mobility and quality of life for amputees.

## 1 Introduction

Due to accidents, vascular disease complications, and wars, hundreds of thousands of individuals worldwide undergo lower-limb amputation each year, resulting in the loss of part or all of their lower limbs and, consequently, their ability to walk. According to the World Health Organization, an estimated 1.3 billion people globally live with significant disabilities, accounting for approximately 16% of the world’s population. Data from China’s 7th National Population Census and the 2nd Disability Sampling Survey reveal that over 26 million people in China have physical disabilities, including more than 1.7 million lower-limb amputees (National Bureau of Statistics of China, 2021; McDonald et al., 2021; Watson et al., 2019). Hip disarticulation, the highest level of lower-limb amputation, accounts for about 2.2% of all lower-limb amputations (McDonald et al., 2021; Watson et al., 2019). Physical disabilities significantly impact the lives of amputees, imposing a considerable burden on their families and society. Thus, research on rehabilitation devices for individuals with disabilities carries both social and scientific significance.

Prosthetics are the primary means of restoring walking functionality in lower-limb amputees. The hip and knee joints are essential for a natural human gait. However, current research on lower-limb prostheses mainly focuses on amputees at or below the thigh level, with limited attention given to prosthetic hips. Existing commercial prosthetic hips are purely mechanical and cannot provide the active energy required for positive work movement of the hip joints. Additionally, these devices fail to automatically adjust the joint torque primarily damping torque according to the amputee’s intentions. Moreover, because existing prosthetic hips cannot actively coordinate with downstream joints, amputees must rely on compensatory movements, such as trunk leaning or hip hiking, to adapt the motion of the prosthetic knee and foot, often resulting in poor hip-knee coordination. Many hip-disarticulation amputees, due to the difficulty and high energy cost of walking with prosthetic devices, choose to abandon their prosthetic legs and rely instead on mobility aids such as wheelchairs (Spence et al., 2001). This underscores that current prostheses are insufficient to meet the needs of these amputees (Luo et al., 2023; Luo et al., 2024).

This paper reviews the research progress in key technologies for intelligent prosthetic hips and knees, with a focus on actuation, perception, and control. The actuation aspect involves utilizing intelligent bionic technologies to design the actuation mechanisms of the prosthesis, enabling the prosthetic joint to replicate the mechanical characteristics and driving/damping compensation mechanisms of the human joint. The perception aspect centers on establishing human-machine-environment interaction channels through the fusion of signals from the human body and the prosthetic device, facilitating motion intention recognition and hybrid decision-making in human-machine collaborative tasks. The control aspect focuses on adjusting prosthesis movements or actuation strategies based on intention recognition results, allowing the prostheses to more closely mimic normal human gait characteristics in dynamically changing environments.

To ensure transparency and reproducibility, this article adopts a systematic review methodology. A structured search was conducted across IEEE Xplore, PubMed, Web of Science, and SpringerLink for studies published between 2010 and 2024, using keyword combinations related to intelligent prostheses, hip/knee actuation, perception, and control. After removing duplicates, titles and abstracts were screened according to predefined inclusion criteria (relevance to prosthetic hip/knee systems, experimental or methodological contribution, and peer-reviewed status), while excluding non-prosthetic, purely theoretical, or methodologically incomplete studies. A two-stage full-text review by two independent evaluators identified 162 eligible papers, which were categorized into actuation, perception, and control domains. From these, we selected 100 representative studies for detailed analysis, supplemented by four seminal works published before 2010. This systematic process ensures the consistency, comprehensiveness, and traceability of the reviewed literature.

## 2 Actuation mechanisms of prosthetic hips and knees

**Figure 1:**
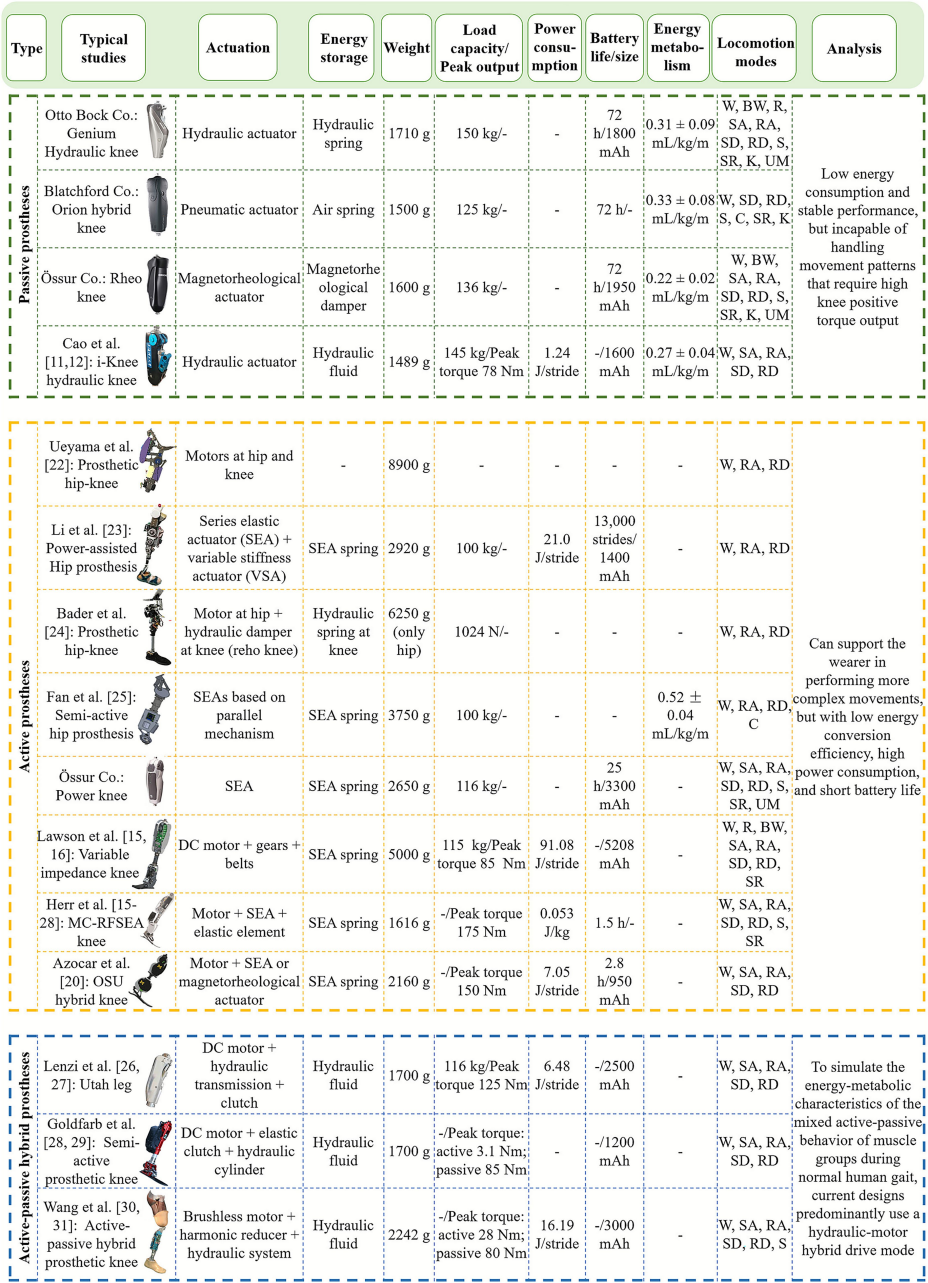
Summary of typical prosthetic hip/knee actuation mechanisms. BW, Backward walking; C, Cycling; K, Kneeling; S, Sitting; SA, Stair ascent; SD, Stair descent; SR, Stumble recovery; R, Running; RA, Ramp ascent; RD, Ramp descent; UM, User-defined mode; W, Level walking.

The hip and knee joints are the most critical and complex components of intelligent lower-limb prostheses. The bionic design of actuation compensation mechanisms that closely replicate human joint functionality is fundamental for achieving effective amputee-prosthesis coordination. Traditional intelligent lower-limb prostheses can be classified into active and passive types based on their ability to generate active torque. In recent years, some researchers have begun exploring hybrid active-passive prosthetic knees (see Figure 1).

### 2.1 Passive prosthetic hips/knees

Passive lower-limb prostheses generate damping torque using pneumatic, hydraulic, and magnetorheological (MR) dampers. By controlling these dampers, joint torque can adaptively adjust to changes in walking speed and gait phases, resulting in a gait that closely resembles that of a healthy individual. Pneumatic and hydraulic dampers operate on similar principles, regulating the flow rate of fluids to control damping. Due to the compressibility of air, pneumatic dampers provide limited damping force, which makes it challenging to maintain stability during the stance phase. MR dampers adjust the viscosity of MR fluids by controlling the current, thereby modulating the output damping force. However, their application is limited due to susceptibility to magnetic field interference. Hydraulic dampers, on the other hand, benefit from the incompressibility of liquids, allowing for high damping forces within a small volume, which ensures stability during the stance phase. Additionally, hydraulic damping exhibits distinct properties depending on the fluid flow rate: linear damping at low flow rates (laminar flow), and nonlinear damping at high flow rates (turbulent flow). By altering the fluid passage area to modify the flow rate and induce turbulence, rapid damping response and control can be achieved.

Currently, passive intelligent prosthetic knees have relatively mature commercialized products globally (Chin et al., 2006; Bellmann et al., 2010). However, there are relatively few research institutions still researching pure passive knees. For example, Liu et al. (2022) and Assfaw & Seid (2022) optimized MR damper designs using B-spline curves and damping force/displacement control parameters, respectively. Wang et al., 2023 and Cao et al. (2018) developed two generations of intelligent hydraulic prosthetic knees and proposed various structures for electro-hydraulic dampers. In contrast, purely passive prosthetic hips have received little attention due to their inability to meet the predominantly positive work requirements of the human hip joint during most gait phases. The main advantages of passive lower-limb prostheses are low energy consumption and stable performance. However, they cannot provide active torque to accommodate gait patterns and phases that require high joint torque output.

### 2.2 Active prosthetic hips/knees

Active lower-limb prostheses primarily generate active torque through actuators such as motors and pneumatic muscles. Pneumatic muscles drive the prosthesis by controlling the injection of high-pressure air; however, their practical application is limited by low energy efficiency and poor portability. Motors, which offer higher energy efficiency compared to pneumatic muscles, are currently the most widely used actuation method in active lower-limb prosthetic joints. Over the past decade, active prosthetic knees have been extensively studied (Fu et al., 2023; Culver et al., 2022; Lawson et al., 2014; Lawson et al., 2014; Rouse et al., 2014; Carney et al., 2019; Shultz et al., 2014; Azocar et al., 2020; Cortino et al., 2024). For example, Herr et al. proposed the Clutchable Series-Elastic Actuator (CSEA) and the Moment-Coupled Rotary-Flexible Series-Elastic Actuator (MC-RFSEA) for powered prosthetic knees (Lawson et al., 2014; Lawson et al., 2014; Rouse et al., 2014; Carney et al., 2019). In recent years, active prosthetic hips have also gained research attention (Luo et al., 2024; Cao et al., 2018; Fu et al., 2023; Culver et al., 2022; Lawson et al., 2014; Lawson et al., 2014; Rouse et al., 2014; Carney et al., 2019; Shultz et al., 2014; Azocar et al., 2020; Cortino et al., 2024; Ueyama et al., 2020; Li et al., 2025; Bader et al., 2023; Fan et al., 2022). Ueyama et al. (2020) developed a motor-driven hip disarticulation prosthesis featuring high-torque motors at both the hip and knee joints. However, its bare weight of over 11 kg did not reduce amputees’ energy expenditure. Li et al. (2025) further developed a power-assisted hip disarticulation prosthesis using a remote-center-of-motion (RCM) mechanism to accurately reproduce the anatomical hip rotation center. The actuator combines series elastic actuator (SEA) with a variable stiffness actuator (VSA), providing lightweight assistance and adjustable compliance. Bader et al. (2023) proposed an integrated hip-knee lower-limb prosthesis that combines an active hip with a commercially available passive knee (Reho Knee, Ossur) via an adjustable chassis mechanism. Fan et al. (2022) proposed a semi-active hip prosthesis based on a parallel-mechanism structure, driven by independent motors for flexion/extension and abduction/adduction. The compact linkage arrangement improves torque efficiency while maintaining a small structural envelope, enabling more natural multi-DOF hip movement. Although active lower-limb prostheses can generate high joint torque to support diverse gait patterns, their disadvantages, such as high power consumption, short battery life, and significant operational noise, make them challenging to implement in practical applications.

### 2.3 Active-passive hybrid prosthetic hips/knees

**Figure 2:**
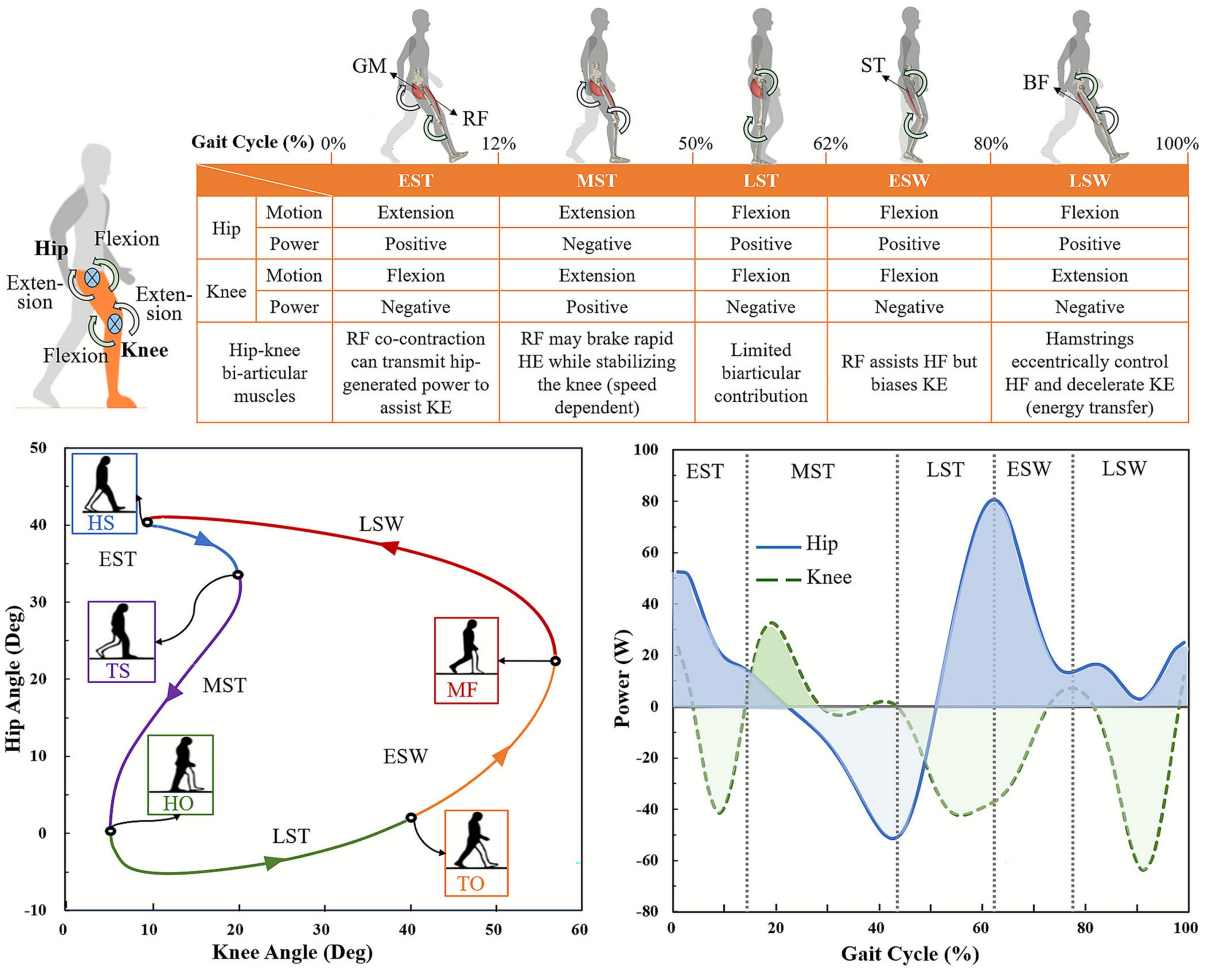
Normal gait patterns of able-bodied individuals during level-ground walking. EST, Early Stance; MST, Mid-Stance; LST, Last Stance; ESW, Early Swing; LSW, Last Swing; GM, Gluteus Maximus; RF, Rectus Femoris; ST, Semitendinosus; BF, Biceps Femoris; HE, Hip Extension, HF, Hip Flexion; KE, Knee Extension; KF, Flexion.

Normal human gait patterns demonstrate that the hip and knee joint muscles employ a hybrid mechanism of active and passive functions, providing active driving torque or passive damping torque during different phases of the gait cycle. Based on the variations in hip and knee angles and power throughout a typical gait cycle during level-ground walking (see Figure 2), there are two primary phases of positive work at the hip joint. The first occurs in early stance when the hip extensor muscles actively drive hip extension, and the second happens in last stance and early swing when the hip flexor muscles actively drive hip flexion. During mid-stance, the primary driving force for hip extension comes from the natural forward shift of the body’s center of mass, requiring the hip muscles to provide damping torque to ensure joint stability and support. For the knee joint, the muscles mainly perform negative work to cushion ground impact and decelerate lower-limb motion. However, during mid-stance, the knee joint must generate a primary torque to facilitate the transition from knee flexion in early stance to full extension in last stance, ensuring effective propulsion.

Based on the gait-phase characteristics shown in Figure 2, different sections of the gait cycle impose distinct requirements on prosthetic actuation. During early stance, substantial positive hip extension torque is required, an area where passive mechanisms often underperform. Mid-stance stabilization relies heavily on damping modulation to prevent excessive collapse of the limb, revealing limitations in systems with slow-response damping control. Last stance and early swing require timely positive hip flexion work, which many existing prosthetic hips cannot provide. For the knee joint, early stance demands effective negative work for shock absorption, while mid-stance requires controlled extension torque that hybrid mechanisms can support more effectively. These phase-specific requirements highlight both the performance strengths and limitations of current actuation approaches, guiding the motivation for hybrid active-passive designs. In recent years, several researchers have started exploring hybrid active-passive prosthetic knee joints (Lenzi et al., 2018; Bartlett et al., 2022; Lee & Goldfarb, 2021; Wang et al., 2023; Wang et al., 2020; Li et al., 2023). For examples, Minh et al. (2022) and Lenzi et al. (2018) proposed a prosthetic knee based on a multi-source actuator, which provides active driving torque during stair ascent but only offers hydraulic damping for other gait modes, without adaptive damping adjustment. Bartlett et al. (2022) and Lee & Goldfarb (2021) designed a hybrid prosthetic knee integrating a multi-chamber hydraulic cylinder and a linear actuation system to assist as needed during the swing phase. Wang et al. (2023) and Wang et al. (2020) proposed an intelligent prosthetic knee with a hydraulic-motor hybrid actuation system, capable of delivering active torque during the stance extension phase of level-ground walking, stair and ramp ascent, while adaptively adjusting damping torque in real time for other phases.

Moreover, energy transfer among lower-limb joints is not independent; instead, it follows a natural energy-saving mechanism through biarticular muscle bidirectional actuation and inter-joint energy flow. Since the proximal and distal attachment points of biarticular muscle-tendon units span two joints, they provide bidirectional actuation, playing a crucial role in energy transfer and recovery during human motion (Wells, 1988; Schenau, 1989). For example, the semitendinosus (ST) and biceps femoris (BF) muscles, which connect the hip and knee joints, store energy through elongation in last swing. This process not only provides damping torque to decelerate shank swing at the knee joint but also generates hip extension torque at the hip joint. This bidirectional actuation mechanism allows partial recovery of swing-leg kinetic energy while assisting hip extension. Currently, few research has focused on hybrid active-passive prosthetic hips. Furthermore, the development of a hybrid actuation mechanism that enables energy flow and recovery between the hip and knee joints remains an open area of research, which could potentially become a future research direction for investigators in this field.

## 3 Human lower-limb motion intention recognition

**Figure 3:**
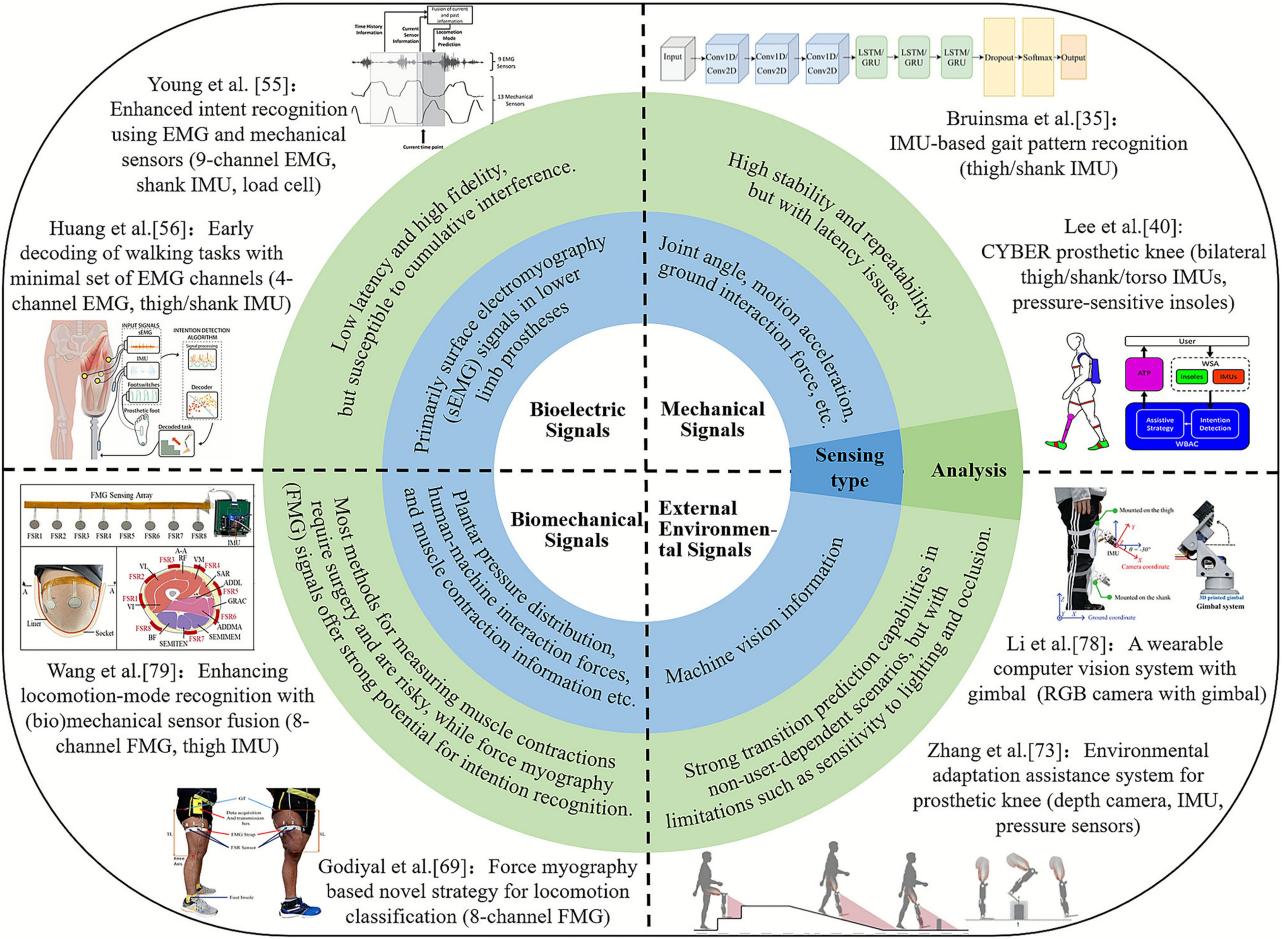
Summary of typical human lower limb motion intention recognition methods.

The recognition of human lower-limb motion intentions can be categorized into four types based on the source of the signals: (1) Mechanical signals: These include physical information reflecting kinematic and dynamic characteristics; (2) Bioelectric signals: The most commonly used in lower-limb prosthetics are electromyography (EMG) signals; (3) Biomechanical signals: These include biological information that reflects the motion state of the human body; (4) External environmental signals: These reflect changes in the surrounding environment and its characteristic parameters (see Figure 3). A typical study on pattern recognition of lower limb prostheses and a comparison of their performance is shown in Table 1.

**Table 1:** Typical studies on intelligent perception of lower limb prostheses.

Type	Typical studies	Sensor used	Locomotion	Transition	Classifier	Accuracy			TPT /TPP	ET	Analysis
						Steady mode	Transition	Overall			
Mechanical signals	Bruinsma & Carloni (2021)	IMU	LW, SA, SD, RA, RD, SIT, STA	Yes (24 trans.)	Long Short-Term Memory (LSTM)	No	No	93.06%	−33.70 ms/No	17.33 ms	High stability and reliability, but with inherent latency
	Su et al. (2019)	IMU	LW, SA, SD, RA, RD	Yes (8 trans.)	Convolutional Neural Network (CNN)	92.20%	88.75%	89.23%	No	No	
	Marcos Mazon et al. (2022)	IMU	LW, SA, SD, RA, RD, SIT, STA	Yes (12 trans.)	CNN-LSTM	No	No	95.0%	−41.88 ms/No	37.09 ms	
	Zhang et al. (2024)	IMU, Load cell	LW, SA, SD, RA, RD, STA	Yes (10 trans.)	Extreme Learning Machine (ELM)	96.75%	No	No	−2 ms/No	2 ms	
	Zhang et al. (2023)	IMU	LW, SA, SD, RA, RD	No	Improved K-Nearest Neighbor (KNN)	96.66%	No	No	9.8 ms/No	No	
	Lee et al. (2021)	IMU, pressure-sensitive insole	LW, SA, SD, RA, RD	No	Bidirectional LSTM (BiLSTM)	No	No	98.33%	-30 ms/No	No	
Bioelectric (−mechanical fusion) signals	Young et al. (2014)	EMG, IMU	LW, SA, SD, RA, RD	Yes (8 trans.)	Dynamic Bayesian Network (DBN)	99.0%	87.8%	No	11.6 ms/No	No	Low latency and high fidelity, but interference-prone
	Barberi et al. (2023)	EMG, IMU	LW, SA, SD, RA, RD	No	Support Vector Machine (SVM)	94.60%	No	No	No/20%	No	
	Peng et al. (2020)	EMG, IMU	LW, SA, SD, RA, RD	Yes (8 trans.)	Linear Discriminant Analysis (LDA)-Random Forest (RF)	97.80%	83.90%	No	14.3 ms/No	No	
External environmental (−mechanical fusion) signals	Zhang et al. (2021)	Depth camera, IMU	LW, SA, SD, RA, RD, OS	Yes (8 trans.)	CNN	No	No	97.34%	No/Esti-mated 70%	23 ms	Strong transition prediction in non-user-dependent settings, but sensitive to lighting and occlusion
	Zhong et al. (2022)	GPS, IMU, Camera	Tile, Cement, Brick, Grass, SA, SD	Yes (10 trans.)	Dynamic Bayesian Gated Recurrent Unit Network	No	No	93.00%	956 ms/No	44 ms	
	Li et al. (2023)	RGB camera with gimbal	LG, SA, SD	Yes (4 trans.)	CNN	98.29%	97.27%	98.03%	2.92 ms/No	67.02 ms	
Biomechanical (−mechanical Fusion) signals	Godiyal et al. (2018)	FMG	LW, SA, SD, RA, RD	No	LDA	96.10%	No	No	No	No	Enables decoding of voluntary human intent with low latency.
	Wang et al. (2025)	FMG, IMU	LW, SA, SD, RA, RD	Yes (8 trans.)	CNN-BiLSTM	99.34%	97.82%	98.51%	274 ms/ 21.82%	7.86 ms	

LW, level walking; SA, stair ascent; SD, stair descent; RA, ramp ascent; RD, ramp descent; SIT, sitting; STA, standing; TPT, transition prediction time; TPP, transition prediction percentage; ET, execution time. Positive values of TPT indicate advanced prediction; Negative values of TPT indicate delayed prediction.

### 3.1 Mechanical signals for intention recognition

Mechanical signals are currently the most widely used in recognizing lower-limb motion intentions, including joint angles, joint torques, motion accelerations, and ground reaction forces, etc. These signals offer advantages such as high stability and repeatability, and existing commercial lower-limb prosthetic products rely on them for motion intention recognition (Bruinsma & Carloni, 2021; Su et al., 2019; Marcos Mazon et al., 2022; Zhang et al., 2024; Zhang et al., 2023; Lee et al., 2021; Zheng et al., 2024). However, motion mechanical signals have inherent time delays, and issues such as mechanical backlash can exacerbate these delays. Additionally, motion mechanical signals typically reflect the motion of the prosthetic device itself rather than the amputee’s autonomous motion intentions. As a result, they can only classify actions that have already occurred and cannot directly map an amputee’s intentions to movements. Therefore, relying solely on motion mechanical signals for human motion intention recognition has unavoidable limitations.

### 3.2 Bioelectric signals for intention recognition

**Figure 4:**
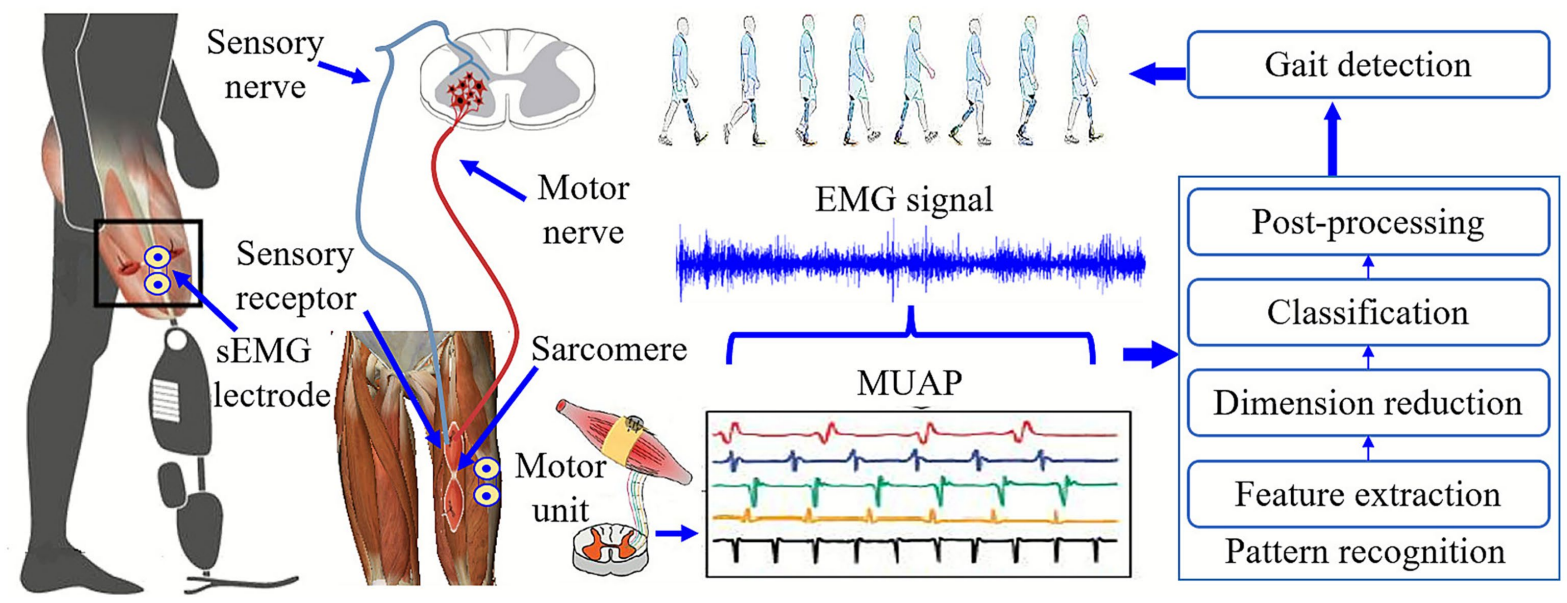
Locomotion intention recognition of above-knee amputees based on sEMG signal (supervisory EMG control).

Bioelectric signals, particularly electromyography (EMG), reflect the activity of the central nervous system and can be obtained invasively or non-invasively. For lower-limb amputees, voluntary muscle contractions provide information from which prosthetic systems extract features, classify locomotion modes, and execute the corresponding control. Thus, EMG-based intention recognition is often referred to as supervised myoelectric control (Fleming et al., 2021), typically involving feature extraction, optional dimensionality reduction, pattern classification, and post-processing (see Figure 4). Valle et al. (2021) proposed an implanted neural-interface system, but invasive techniques cause tissue damage and remain unsuitable for widespread clinical use. Non-invasive sEMG offers low latency, high fidelity, and easy acquisition, and has been used extensively in upper-limb prosthetics; however, its application in lower-limb prosthetics remains limited. Current research mainly focuses on sensor design, feature selection, and classification methods (Fleming et al., 2021).

sEMG sensor design must address challenges such as socket-residual-limb interface pressure, sweat accumulation, and limb-volume changes. Most studies place bipolar electrodes inside the socket liner (Clites et al., 2018; Shu et al., 2021; Huang & He, 2018), but this may impair suspension and comfort, limiting long-term use. Embedded-electrode liners offer one solution (Rogers et al., 2021), though they require custom fabrication. Yeon et al. (2021a) Yeon et al. (2021b) developed flexible electrodes compatible with existing sockets, achieving signal quality comparable to commercial sensors. Accurate electrode placement is another challenge due to residual muscle atrophy and reduced activation. Some researchers enhance signal availability using targeted muscle reinnervation (TMR); Pasquina et al. (2021) showed that transferring transected nerves to healthier muscles can improve voluntary contraction, though its surgical risks remain uncertain.

Feature selection and classification strategies are also critical. Studies have used time-domain (e.g., zero crossings, mean absolute value) and frequency-domain features (e.g., median frequency) for mode classification (Hussain et al., 2020), with dimensionality-reduction methods such as PCA helping to prevent overfitting (Spanias et al., 2018). Classifiers commonly include artificial neural networks, support vector machines, discriminant analysis, and Bayesian models. Innovations in dimensionality reduction, artifact suppression, and classifier design continue to improve recognition accuracy (Gupta & Agarwal, 2019; Dhindsa et al., 2022; Young et al., 2014; Barberi et al., 2023; Peng et al., 2020). Nevertheless, sEMG has not become a widely adopted sensing modality for lower-limb prostheses because its signals are not highly stationary across gait cycles and their mapping to limb motion exhibits considerable variability and uncertainty (Kristjansson et al., 2017; Tschiedel et al., 2020).

### 3.3 Biomechanical signals for intention recognition

To more effectively extract human motion information, some researchers have utilized biomechanical data, such as muscle contraction signals during walking, for motion intention recognition in lower-limb amputees. A notable example is the Magnetomicrometry technology proposed by Taylor et al. (2021) and Taylor et al. (2022), which tracks muscle contraction velocity by measuring the relative motion of magnetic beads implanted in muscles. Another example is the non-contact capacitive sensing method for motion pattern recognition in thigh amputees, proposed by Wang et al. (2019) and Xu et al. (2020). However, both methods require surgical implantation of sensing components, raising concerns about safety and potential long-term postoperative complications.

In recent years, researchers have explored the use of force myography (FMG) signals for human motion intention recognition. FMG signals refer to pressure changes at the human-machine interface caused by dimensional changes in muscles during contraction or relaxation, particularly when external materials spatially constrain the muscles during motion (Godiyal et al., 2018). FMG measurement is non-invasive, cost-effective, and less affected by factors such as skin impedance changes compared to sEMG signals, offering greater stability (Zheng et al., 2022). These characteristics make FMG a promising signal source for motion intention recognition. FMG signals have been widely applied in upper-limb motion classification, such as gesture recognition (Zheng et al., 2022; Asfour et al., 2021), but their application in lower-limb intention recognition remains in the early stages (Zou et al., 2022). For example, Kwak et al. (2020) developed a socket with integrated flexible pressure/temperature sensors capable of continuously monitoring pressure and temperature within the socket but did not utilize the pressure signals for amputee intention recognition. Godiyal et al. (2018) used thin-film pressure sensors to measure FMG signals from the residual limbs of thigh amputees and classified five terrain conditions using linear discriminant analysis. However, real-time intention recognition was not achieved, and the classification accuracy needs improvement. Thus, FMG signals hold significant potential for motion intention recognition in lower-limb amputees, with substantial opportunities for development in FMG feature extraction and direct mapping to joint movements.

### 3.4 External environmental signals for intention recognition

External environmental signals can predict human motion intentions in advance based on surrounding terrain information, enhancing the gait adaptability of prosthetic users in complex environments (Tschiedel et al., 2020). Commonly used external environmental perception sensors (referred to as external sensors) in lower-limb prosthetics include laser rangefinders (Luo et al., 2023), ultrasonic distance sensors (Sahoo et al., 2020), and depth cameras (Al-Dabbagh & Ronsse, 2022; Zhang et al., 2021). One challenge for external sensors is visual interference caused by the amputee’s body motion during walking. Some studies have placed sensors at higher positions on the body, such as the waist or chest (Krausz & Hargrove, 2021; Garrett et al., 2022; Brokoslaw et al., 2022), which reduces system integration and wearing comfort. Additionally, a broader field of view can introduce more irrelevant image data, interfering with prediction performance. Another common approach is to attach external sensors directly to the prosthetic limb to improve system integration (Sahoo et al., 2020; Zhang et al., 2021; Zhong et al., 2022). However, lower-limb movements (e.g., heel strikes and swing flexion) often result in blurred images and a narrowed field of view, negatively impacting recognition accuracy. To ensure sensing stability, most related studies only support forward terrain recognition during the mid-stance phase (Li et al., 2023). In summary, while external environmental perception signals are predictive, relying solely on these signals can lead to motion intention misjudgments and incorrect mode transitions. Minimizing interference to external environmental perception systems mounted on prosthetic limbs during dynamic human-machine-environment interactions remains an unresolved challenge.

### 3.5 Intention recognition based on multi-source information fusion

**Figure 5:**
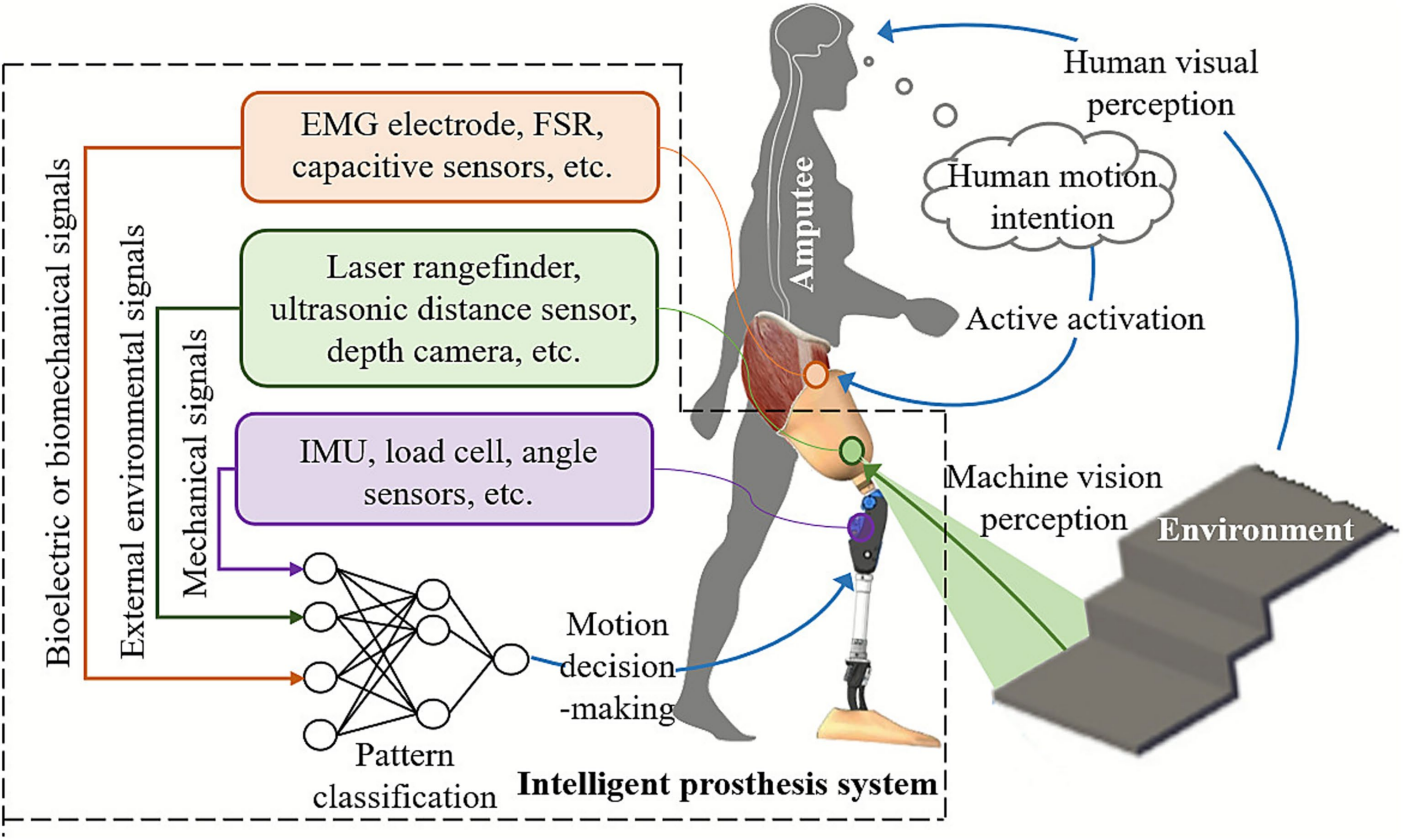
An example of locomotion intention recognition of above-knee amputees based on multi-source information fusion.

Relying solely on a single type of signal for human motion-intention recognition presents unavoidable limitations. For example, myoelectric signals raise safety and robustness concerns, mechanical signals suffer from inherent delays, and environmental-perception signals are prone to misclassification. These factors make it difficult to achieve ideal recognition performance across varying terrains, walking speeds, and gait phases (Tschiedel et al., 2020). Therefore, fusing biomechanical signals, myoelectric signals, and environmental-perception data—so that each modality compensates for the weaknesses of the others—has become an important research direction for achieving higher accuracy, stronger real-time performance, and more effective human-machine-environment interaction (see Figure 5). For instance, Wang et al. (2025) developed a dual-modal FMG-IMU sensing system for powered prosthetic knees, in which fused FMG and inertial signals were classified using a CNN-BiLSTM model. This approach yielded high locomotion-mode recognition accuracy and effective transition prediction, demonstrating that FMG combined with additional sensing modalities can support reliable real-time gait-intention detection. Zhong et al. (Li et al., 2023) proposed an environment-prediction framework for powered lower-limb prostheses based on IMU, camera, and GPS sensing. By employing an uncertainty-aware model-selection strategy that uses lower-limb kinematics and Bayesian-network-estimated environmental uncertainty, the system dynamically selects the optimal prediction module, thereby improving computational efficiency.

## 4 Intelligent control of prosthetic hips and knees

**Figure 6:**
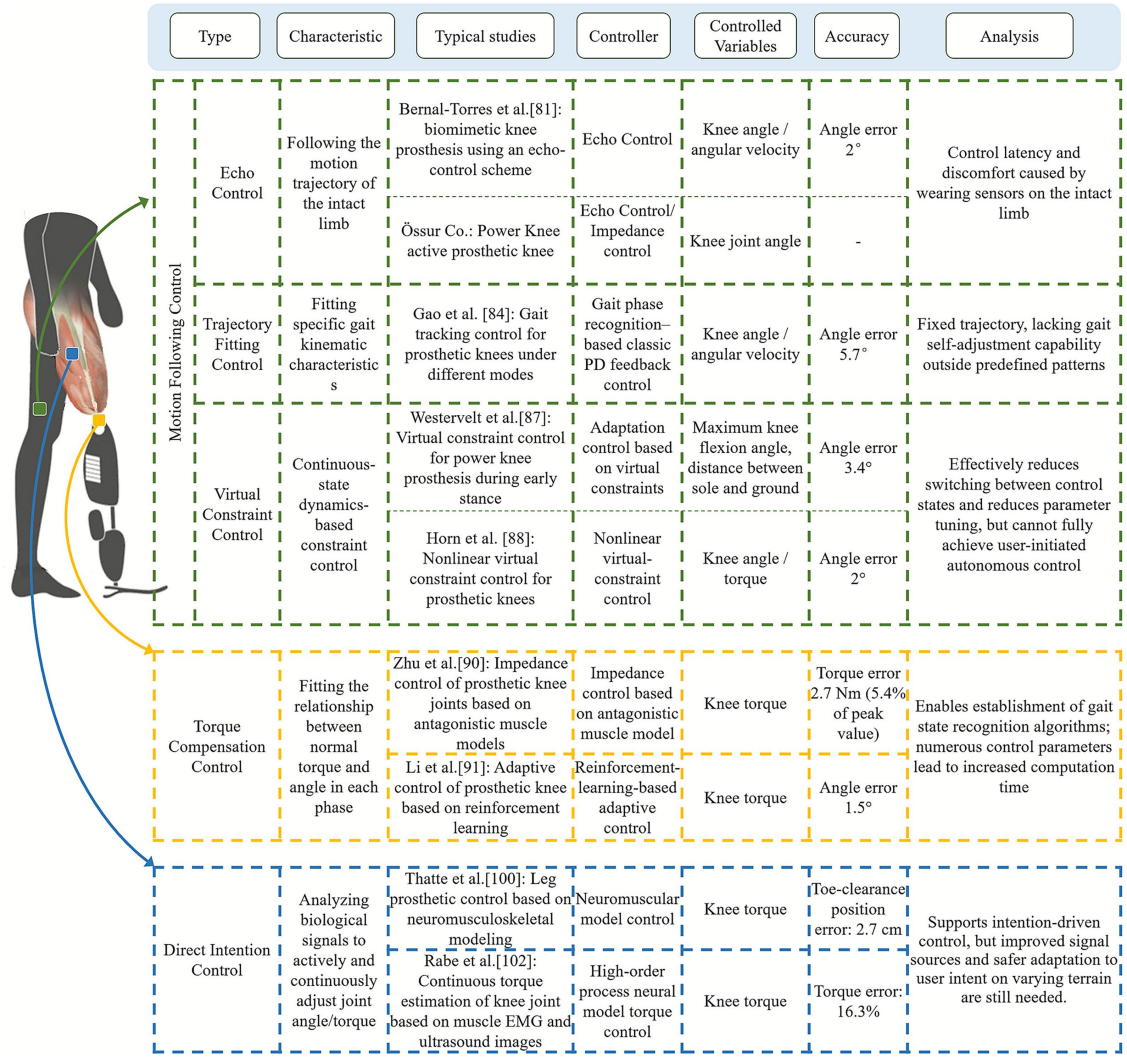
Summary of typical intelligent control methods for prosthetic hips and knees.

Based on different control objectives, intelligent control methods for lower-limb prosthetics can be categorized into three main types: (1) Torque compensation Control: calculating the ideal joint torque based on the amputee’s walking state, enabling the prosthetic to achieve regulated motion; (2) Motion following Control: ensuring prosthetic motion follows predefined kinematic trajectories; (3) Direct intention Control: mapping the amputee’s biological signals to joint angles or torques, allowing the prosthetic to actively and directly adjust to align with the amputee’s motion intentions (see Figure 6).

### 4.1 Motion following control

Motion following control primarily includes mapping control (also known as echo control), trajectory fitting control and virtual constraint control. Mapping control is one of the earliest control strategies applied to lower-limb prosthetics. This method records the motion trajectory of the intact limb using sensors and replicates it on the prosthetic side, ensuring good gait symmetry (Yang et al., 2022). For examples, the active hip-disarticulation prosthesis by Ueyama et al. uses this approach (Ueyama et al., 2020), and Bernal-Torres et al. (2018) developed a variable-center prosthetic knee that converts intact-side knee angles into motor commands for gait following. Although effective for symmetry, mapping control suffers from transmission delays, the need for intact-side sensors, and reduced adaptability beyond steady-state walking.

Trajectory fitting control assigns predefined joint trajectories to each locomotion mode, triggered by intention-recognition outputs, thereby simplifying control design (Huang et al., 2022; Alili et al., 2023). Gao et al. (2019) classified EEG signals and commanded prosthetic joints to follow ground-reaction-force-encoded trajectories.

The limitation is that the method relies on fixed reference trajectories, which work best during rhythmic walking but are less effective during transitions.

Virtual constraint control, originally developed for bipedal robots, enforces joint trajectories defined by phase variables derived from lower-limb kinematics and has recently been applied to prosthetic devices (Li et al., 2025; Sullivan et al., 2025; Lv et al., 2024; Westervelt et al., 2018; Horn & Gregg, 2021). Mendez et al. (2020) proposed a powered knee-ankle prosthesis that dynamically adjusts swing-phase trajectories for obstacle negotiation based on residual-limb motion. While effective, reliance on mechanical signals introduces latency, which limits smoothness in rapid adjustments. Virtual constraint control shows promising clinical potential, though its compatibility across multiple locomotion modes requires further validation.

### 4.2 Torque compensation control

Torque compensation control is primarily implemented through impedance control based on a finite state machine (FSM). This strategy models the joint as a virtual spring-damper system, using piecewise impedance functions to approximate the torque-angle relationship in each gait phase. During walking, gait events are first detected through an intention-recognition algorithm, which determines the appropriate control model. FSM-based impedance control is widely used in active lower-limb prosthetics (Azocar et al., 2020; Zhu et al., 2022; Li et al., 2021; Wu et al., 2022; Heins et al., 2021; Elery et al., 2020). For example, Zhu et al. (2022) developed a back drivability-based knee control method capable of predicting and tracking knee torque in transfemoral amputees, while Li et al. (2021) proposed a reinforcement-learning impedance strategy for personalized torque assistance, although it operated only at several discrete knee positions rather than across the full gait cycle.

Despite its prevalence, torque compensation control has limited adaptability, as proportional-derivative gains and switching rules must be carefully tuned for each user, activity, and gait phase. Although online optimization algorithms have been introduced, the large number of parameters still burdens the control system. Moreover, although Liu et al. (2014) improved phase-transition accuracy using a Dempster-Shafer-based rule, achieving precise timing and smooth, seamless transitions across gait modes remains a major challenge (Alili et al., 2023).

### 4.3 Direct intention control

**Figure 7:**
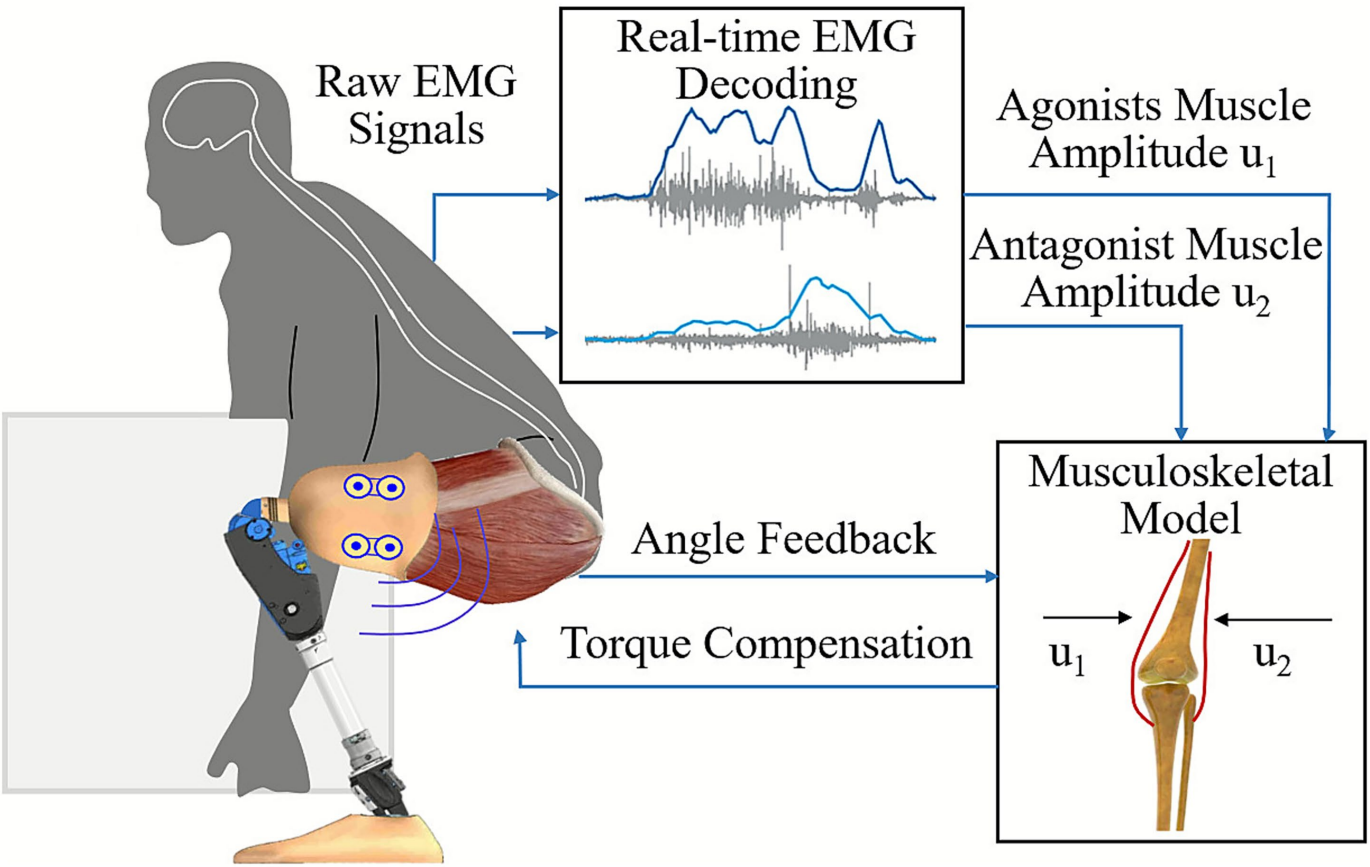
An example of direct EMG control (Fleming et al., 2021).

The motion trajectory following and torque compensation control methods commonly used in intelligent lower-limb prosthetics can only guide the prosthesis to follow the intact-limb trajectory or predefined gait-mode trajectories. These approaches cannot incorporate the user’s continuous voluntary enabling intentions into low-level torque or position control, and therefore cannot achieve truly natural gait through direct user-driven parameter modulation. In recent years, studies have suggested that myoelectric signals can be used directly for low-level control. Such direct myoelectric control interprets the wearer’s continuous EMG signals to adjust prosthetic joint impedance, angle, or torque, allowing the mechanical behavior of the prosthetic joint to be determined directly by the user’s feedforward neural output (see Figure 7; Barberi et al., 2023).

Proportional control is one of the earliest methods used in direct intention control for prosthetics (Fleming & Huang, 2019). It maps biological signals and control targets into simple mathematical relationships, leading to inaccuracies in the mapping and low control precision (Fleming et al., 2021). To address this, some researchers have incorporated muscle dynamics from musculoskeletal models into the control systems (Shu et al., 2022). Thatte & Geyer (2015) proposed a neuromuscular-model-based prosthetic control method in which seven Hill-type muscle tendon units are used to represent human musculature. The torque and activation signals generated by the model are converted into motor voltages through an SEA controller to produce joint torques in a bipedal robotic platform. However, the forward dynamic computations required for musculoskeletal models are time-consuming, making it difficult to meet the real-time requirements of lower-limb prosthetic control. Researchers have turned to machine learning to replace musculoskeletal mathematical models, aiming to effectively handle the nonlinear relationships between biological signals and joint movements (Siu et al., 2021). For example, Rabe et al. (2021) used lower-limb sEMG signals and skeletal muscle ultrasound imaging as inputs for a Gaussian process regression model to estimate hip, knee, and ankle joint torques under different movement modes.

## 5 Development trends in intelligent prosthetic hip-knee

### 5.1 Hip-knee coordinated hybrid active-passive compensation actuation

Overall, studies on lower-limb prosthetics have demonstrated that simulating the hybrid active-passive actuation mechanism of lower-limb joint muscles during normal human gait is a necessary trend for reducing the energy consumption of the amputee-prosthesis coupling system. However, research on intelligent lower-limb prosthetics targeting high-level amputees (hip disarticulation) remains very limited. Existing studies primarily adopt a fully active actuation approach throughout the entire gait cycle. Additionally, prosthetic hip and knees are typically designed as integrated systems, which can only simulate the hip-knee coordination relationship through the independent control of the two joints, without enabling energy flow between them. Biomechanical studies have shown that human lower-limb hip-knee joints exhibit a natural bidirectional hybrid active-passive actuation characteristic mediated by biarticular muscles. This natural coordination and energy transfer between joints play a crucial role in gait energy flow and recovery. The key challenge for advancing lower-limb prosthetic research lies in designing bionic structures and actuation mechanisms that establish a bidirectional hybrid active-passive energy compensation system, thereby replicating the natural actuation characteristics of lower-limb muscles and improving energy utilization efficiency.

### 5.2 Human volitional (autonomous) control

Most intelligent prosthetic knee systems currently adopt a hierarchical control framework in which human motion intention and gait phase are first recognized, and low-level control is then executed based on the identified locomotion mode and phase. This approach relies heavily on pre-trained models for each gait mode, resulting in mode-switching delays and non-smooth transitions. Moreover, widely used low-level controllers only enable the prosthesis to follow the intact-limb trajectory or predefined trajectories for specific gait modes. They cannot incorporate the user’s continuous volitional enabling signals into low-level torque or position control, and thus cannot realize truly natural, user-driven gait.

Although direct myoelectric control reflects the electrical activity associated with voluntary muscle contractions, its effectiveness is limited by the inherent non-stationarity and variability of EMG signals (Wang et al., 2023). Emerging sensing modalities—such as capacitive sensing (Wang et al., 2019; Xu et al., 2020), magnetic micro-displacement sensing (Taylor et al., 2021; Taylor et al., 2022), and FMG-based muscle-pressure sensing (Wang et al., 2025)—offer access to physical measurements of muscle contraction (e.g., muscle length and contraction velocity), enabling direct muscle-driven control for interpreting human volitional intent. Such control requires establishing a mapping between muscle contraction information and prosthetic joint targets (position and velocity) using muscle mathematical models or neural-network models. However, major challenges remain. First, residual-limb muscles often exhibit abnormal activation after amputation (Huang & Ferris, 2012), and substantial inter-individual variability complicates the development of robust models. Second, an adaptive decoding mechanism is needed to ensure that amputees can learn to adjust residual-muscle activation when discrepancies arise between the desired and actual prosthesis states.

### 5.3 Human-machine-environment bidirectional closed-loop interaction

Intelligent prosthetic hip/knee systems represent highly integrated human-machine-environment ecosystems in which establishing both physical and cognitive bidirectional interaction pathways is essential for achieving natural gait. It remains an open question whether environmental augmentation and sensory feedback can enhance the performance of direct muscle control in prosthetic knees. Current environmental-augmentation approaches primarily rely on machine vision to predict upcoming terrain, providing prior contextual information for intention recognition and enabling hybrid decision-making in complex environments. However, depth cameras and distance sensors used in prosthetic vision systems face strict constraints because their field of view must remain unobstructed. Sensory feedback, such as joint position, muscle force, or movement cues, can help users perceive terrain, place the foot correctly, and maintain balance. Most existing work relies on non-invasive technologies such as vibrotactile and electrical stimulation, though these methods offer limited information bandwidth and resolution. As mentioned earlier, a recent study involving implanted neural interfaces in amputees demonstrated how integrating neural sensory feedback can influence motor strategies during prosthesis use (Valle et al., 2021). Nonetheless, current invasive sensory-feedback technologies pose direct physical risks and other unknown challenges, and remain far from large-scale clinical application.

## 6 Conclusion

This review has comprehensively analyzed current developments and challenges in intelligent prosthetic hip and knee technologies, focusing on actuation, perception, and control systems. While passive prostheses provide stable and energy-efficient solutions, their limitations in adapting to dynamic gait demands underline the need for active or hybrid solutions. Recent advancements in hybrid active-passive mechanisms offer promising avenues by closely imitating the natural biomechanics and energy transfer facilitated by human biarticular muscles. However, substantial challenges remain in accurately recognizing real-time human motion intentions and adapting prosthetic control strategies dynamically to complex environmental contexts. Future research should prioritize enhancing the stability, accuracy, and real-time capabilities of intention recognition technologies, alongside refining hybrid actuation mechanisms. Such developments will significantly advance the performance and adaptability of intelligent prosthetic hips and knees, ultimately enabling amputees to achieve more natural gait patterns, reduced energy expenditure, and improved overall quality of life.
